# Machine learning prediction model for postoperative outcome after perforated appendicitis

**DOI:** 10.1007/s00423-022-02456-1

**Published:** 2022-02-15

**Authors:** Roman M. Eickhoff, Alwin Bulla, Simon B. Eickhoff, Daniel Heise, Marius Helmedag, Andreas Kroh, Sophia M. Schmitz, Christian D. Klink, Ulf P. Neumann, Andreas Lambertz

**Affiliations:** 1 Department of General, Visceral and Transplantation Surgery, RWTH Aachen University Hospital, Pauwelsstrasse 30, 52074 Aachen, Germany; 2 Department of Surgery, Hospital Linnich, Linnich, Germany; 3 Institute for Systems Neuroscience, Medical School, Heinrich-Heine University, Düsseldorf, Germany; 4 Institute of Neuroscience and Medicine (INM-7) Research Center, Jülich, Germany; 5 Department of Surgery, Diakonissen Hospital Speyer, Speyer, Germany

**Keywords:** Prediction model, Perforated appendicitis, Machine learning, Complicated appendicitis

## Abstract

**Purpose:**

Appendectomy for acute appendicitis is one of the most common operative procedures worldwide in both children and adults. In particular, complicated (perforated) cases show high variability in individual outcomes. Here, we developed and validated a machine learning prediction model for postoperative outcome of perforated appendicitis.

**Methods:**

Retrospective analyses of patients with clinically and histologically verified perforated appendicitis over 10 years were performed. Demographic and surgical baseline characteristics were used as competing predictors of single-patient outcomes along multiple dimensions via a random forest classifier with stratified subsampling. To assess whether complications could be predicted in new, individual cases, the ensuing models were evaluated using a replicated 10-fold cross-validation.

**Results:**

A total of 163 patients were included in the study. Sixty-four patients underwent laparoscopic surgery, whereas ninety-nine patients got a primary open procedure. Interval from admission to appendectomy was 9 ± 12 h and duration of the surgery was 74 ± 38 min. Forty-three patients needed intensive care treatment. Overall mortality was 0.6 % and morbidity rate was 15%. Severe complications as assessed by Clavien-Dindo > 3 were predictable in new cases with an accuracy of 68%. Need for ICU stay (> 24 h) could be predicted with an accuracy of 88%, whereas prolonged hospitalization (greater than 7–15 days) was predicted by the model with an accuracy of 76%.

**Conclusion:**

We demonstrate that complications following surgery, and in particular, health care system-related outcomes like intensive care treatment and extended hospitalization, may be well predicted at the individual level from demographic and surgical baseline characteristics through machine learning approaches.

**Supplementary Information:**

The online version contains supplementary material available at 10.1007/s00423-022-02456-1.

## Introduction

Appendectomy in case of acute appendicitis is one of the most common (emergency) operative procedures worldwide especially not only in children but also in adult patients. During the last two decades, surgical access (open vs. laparoscopic) and indications for conservative versus surgical procedure have been sufficiently discussed [1]. In case of a complicated respectively perforated appendicitis, surgical treatment is still the standard procedure. Within the last 20 years, primary laparoscopic approach has been established even in suspected complicated/perforated appendicitis [2–4]. In this context, the rate of laparoscopic appendectomy in children in the USA increased from 9.9% in 1999 to 46.6% in 2007 [5]. Today, laparoscopic appendectomy has become a standard procedure for acute appendicitis and the benefits of this technique (fewer wound effects, reduced pain after surgery, shorter hospital stay, and earlier return to normal activity) have been demonstrated in several studies and confirmed in meta-analyses [6]. On the other hand, primary open appendectomy is still performed daily in hospitals, especially in suspected complicated cases.

The motivation of this study was to investigate whether the postoperative outcome of new patients with perforated appendicitis is robust predictable albeit high variability in individual outcomes. A clinical implication for the early prediction of the individual outcome can be an earlier transfer of the patient to a higher level of care, e.g., an intensive care unit.

Therefore, the aim of the present study was to provide comprehensive data of clinical outcomes of perforated appendicitis of all patients treated in our center over a 10-year period and on the basis of that to develop and establish a machine learning prediction model for postoperative outcomes of previously unseen, individual patients.

## Materials and methods

Between 2005 and 2015, a total of 163 patients underwent open or laparoscopic operation for perforated appendicitis at the Department of General, Visceral, and Transplantation Surgery and therefore fulfilled the inclusion criteria for this study. The diagnosis of perforated appendicitis was defined as the conclusive combination of intraoperative findings by the surgeon and histopathological confirmation.

All patients received a single dose of antibiotic prophylaxis before skin incision. Appendectomies in the period from 2005 to 2010 were routinely performed as open access surgery. Since 2010, the standard procedure has been changed to a laparoscopic approach via three trocar exploration. Postoperative oral intake and mobilization were conducted following the fast track concept. Our standard operating procedure was to start oral fluids on day 0 when the patient is awake. On day 1 after the operation, we enlarge the oral intake with yoghurt, biscuit, and etc. and mobilization resp. physiotherapy was performed.

We collected basic demographic data (age, gender, height, weight, and body mass index [BMI]), clinical-anamnestic data such as the American Society of Anaesthesiologists (ASA) score, comorbidities (arterial hypertension, coronary heart disease, chronic obstructive pulmonary diseases [COPD], diabetes mellitus), and perioperative data (time interval from admission to appendectomy, operative time, hemoglobin, C-reactive protein, white-cell count, platelets, INR, open surgery, laparoscopic surgery, conversion, extended surgical procedures during appendectomy, drains) as predictor variables (features). Outcome variables (targets) were defined as the duration of postoperative hospital stay, mortality within 30 postsurgical days, intensive care treatment and length of ICU stay, complications classified by Clavien-Dindo, wound infection, and antibiotic treatment after discharge. The present study was conducted in accordance to the principles of the Declaration of Helsinki and “good clinical practice” guidelines. The study was approved by the local ethics committee (EK 127/16).

### Statistical analysis

To assess whether complications may be predicted from clinical and demographic variables in new, individual patients via machine learning, we employed a random forest classifier with stratified subsampling that was evaluated using a replicated 10-fold cross-validation.

First, the entire dataset was divided into 10 equally sized parts, of which 9 were used for training the algorithm. The remaining 10% of the cases that were not seen by the algorithm during the training phase were then used as the test set for evaluation. That is, we employed the algorithm trained on 90% of the data on the unseen 10% in order to test how well the derived classification rules generalized to new data. This procedure was then in turn repeated for all sections of the data so that every case was once part of the test sample. The performance was quantified by the balanced accuracy, i.e., the mean of sensitivity and specificity, providing a measure of prediction accuracy independently of the a priori ratio between the two possible outcomes. To ensure that this evaluation was independent of the initial split when constructing the fold, this procedure was replicated 50 times, resulting in a stable performance assessment.

For the actual learning and subsequent prediction, we employed random forest classification based on stratified undersampling, i.e., an ensemble of decision trees. The key idea of ensemble classifiers is to repeatedly fit a model mapping between the features (clinical and demographic information) and the target variables (outcomes) based on subsets of the training data. These are then combined into a final prediction model allowing to obtain robust and more accurate performance than obtainable from any of the constituent predictors. Moreover, this approach also allowed us to address the rather skewed distribution of some outcomes, i.e., the fact that some complications were relatively rare. This poses a problem to any machine learning algorithm as it biases the learning towards the more likely outcome. To address this challenge, we undersampled the training cases based on their (known) outcomes to yield smaller subsamples that featured the same number of either outcomes. The individual trees, i.e., weak learners, that were later combined to the final model, were then trained on this balanced data and hence free of any systematic bias towards a particular outcome.

For each weak learner, providing a non-linear transformation between the input features and the targets in the balanced subset, we used decision trees estimated by the interaction test for predictor selection. In contrast to standard classification and regression trees (CART), this algorithm chooses the split predictor that minimizes the *p*-value of chi-square tests of independence between each predictor as well as between each pair of predictors. Minimum parent size was set to 6 cases, i.e., the algorithm was only allowed to add new leaves to branches of at least 6 patients in order to control tree complexity and prevent overfitting. The ensuing model could then be applied to predict the clinical outcomes of a “new” patient from the test set, i.e., a case that was not seen when training the model. This prediction is recorded and the procedure repeated 25.000 times with new, independent sampling from the training data, each yielding a new prediction of the test case. These individual predictions are then aggregated, a process termed “bagging,” to yield the final prediction for the test case.

To assess the relevance of each clinical feature for prediction, we estimated unbiased predictor importance values, by summing changes in the risk due to splits on every predictor and dividing the sum by the number of branch nodes, for each individual weak learner. Given that relatively noisy estimation based on the small but unbiased subsamples, predictor importance estimates for each fold were computed as the median of the ensuing values and then averaged across folds and replications. For final display, we retained those importance estimates that cumulatively accounted for 95% of the total relevance scores.

## Results

### Baseline surgical characteristics and perioperative data

A total of 163 patients (71 female; 92 male) were included in the study. Mean age (± standard deviation) of all patients was 38 ± 26 years with a BMI of 23 ± 7 kg/m^2^. A total of 50% of all patients were ASA grade I and none grade IV. Ten patients (6%) had previous abdominal operations and the interval from admission to appendectomy was 9 ± 12 h. Values of WBC count and CRP were elevated at admission with 15.5 ± 5.1 10^9^ WBC/l and 88.8 ± 70.2 mg/l CRP (reference < 5 mg/l). Detailed patient and baseline surgical characteristics are given in Table 1.

**Table 1:** Basic patient characteristics and perioperative data

Parameter	*n* = 163
Age	38.1 ± 26.3
Gender	M: 92 (56%), F: 71 (44%)
Height (cm)	158 ± 24.6
Weight (kg)	61.7 ± 30
BMI	23.4 ± 6.9
ASA	
ASA I	82 (50%)
ASA II	34 (21%)
ASA III	47 (29%)
ASA IV	0 (0%)
Previous abdominal operation	10 (6%)
Diabetes	13 (8%)
COPD	23 (14%)
CHD	37 (23%)
Renal disease	13 (8%)
Neurological disorder	13 (8%)
Admission to surgery time (hours)	9 ± 12
WBC (G/l)	15.5 ± 5.1
CRP (mg/l)	88.8 ± 70.2
Hb (g/l)	139 ± 17
Time of operation (min)	74±38
Laparoscopic approach	64 (39%)
Conversion rate	7 (11% of *n* = 64)
Primary open surgery	99 (61%)
McBurney incision	60 (59% of *n* = 99)
Midline incision	34 (33% of *n* = 99)
Transverse incision	5 (5% of *n* = 99)
Appendectomy	137 (84%)
Cecal resection	18 (11%)
Ileo-cecal resection	8 (5%)
Drainage	143 (91%)

A total of 64 patients underwent laparoscopic surgery, whereas 99 patients got an open procedure. In case of primary open surgery, incision was done at McBurney`s point in 60 cases (59%), and in 33% via midline incision, respectively 5% via transverse incision. The conversion rate from initial laparoscopic to open surgery was 11%. Mean operation time was 74 ± 38 min.

The most common resection was the appendectomy in 137 patients (84%), whereby in 11% a resection of the cecal-pole and in 5% an ileo-cecal resection were necessary. A drain was placed in 143 patients (91%). Detailed characteristics are shown in Table 1.

### Outcome parameter

The in-hospital overall morbidity was 15% and overall mortality was 0.6%. Detailed Clavien-Dindo classification is shown in Table 2.

**Table 2:** Complications/outcome parameter

Parameter	*n* = 163
Intensive care unit	43 (26%)
Days in intensive care unit	1 ± 2
Morbidity	39 (24%)
Clavien 3a	9 (6%)
Clavien 3b	13 (8%)
Clavien 4a	2 (1%)
Clavien 4b	1 (1%)
Clavien 5	1 (1%)
Pneumonia	5 (3%)
Pulmonary embolism	0
Revision-OP	16 (10%)
Open abdomen	2 (1%)
Surgical site infection	27 (16%)
Fascial dehiscence	3 (2%)
Insufficiency of the stump	3 (2%)
Postoperative stay	9 ± 6
Antibiotics at discharge	17 (10%)

After the operation, 43 patients (26%) received intensive care therapy for 1 ± 2 days. Sixteen patients (10%) received a revision operation, mostly planned lavage (11 patients) due to initial peritonitis. Wound infection rate was 17% and 3 patients (2%) got a fascial dehiscence with need for an open abdomen in 2 patients (1%). Insufficiency of the stump occurred in 3 patients (3%). Mean hospital stay was 9 days (± 6 days) and after discharge 17 patients (10%) continued oral antibiotics (Table 2).

### Prediction model

**Figure 1:**
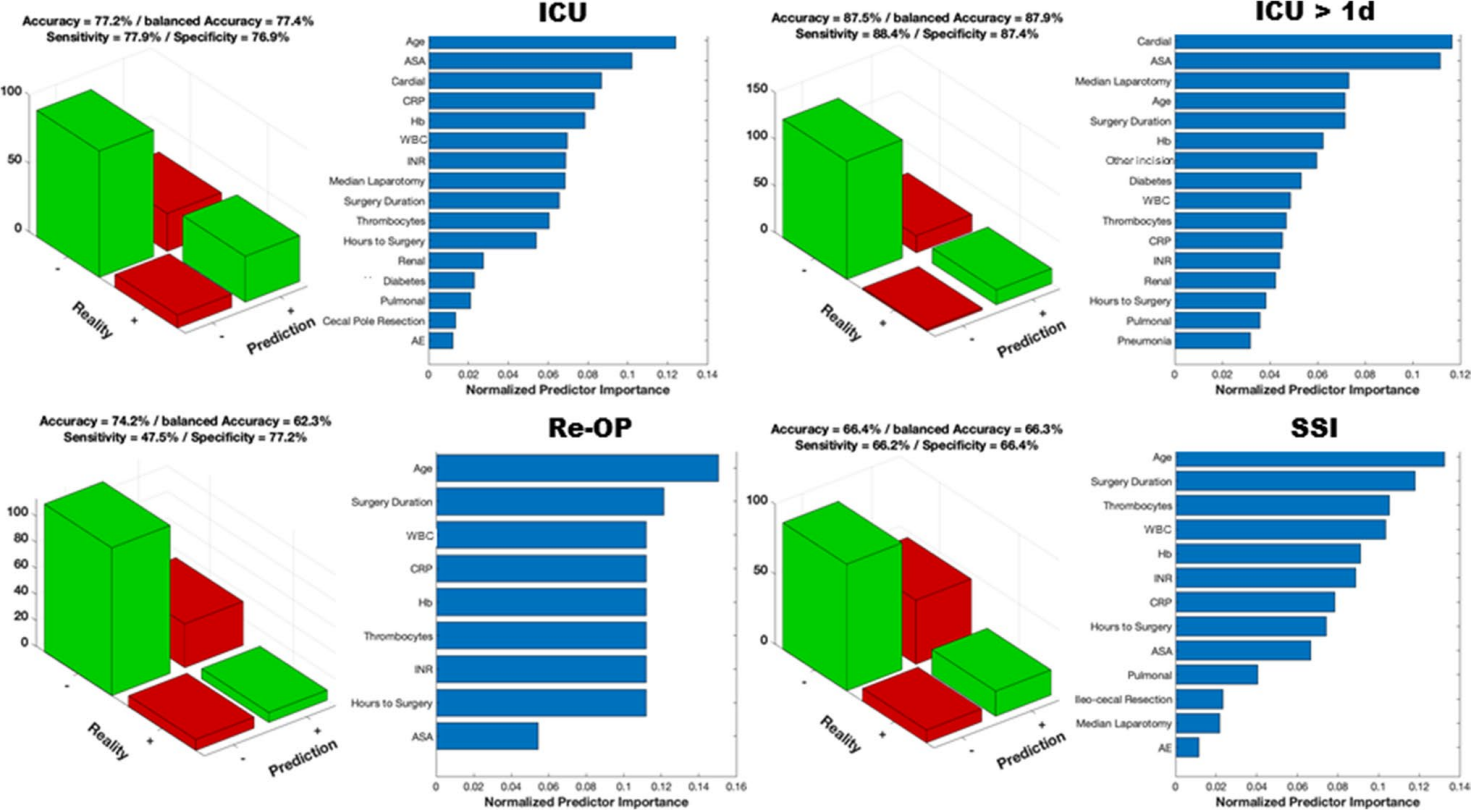
Fourfold table outcome parameter (ICU, Re-OP, SSI)

**Figure 2:**
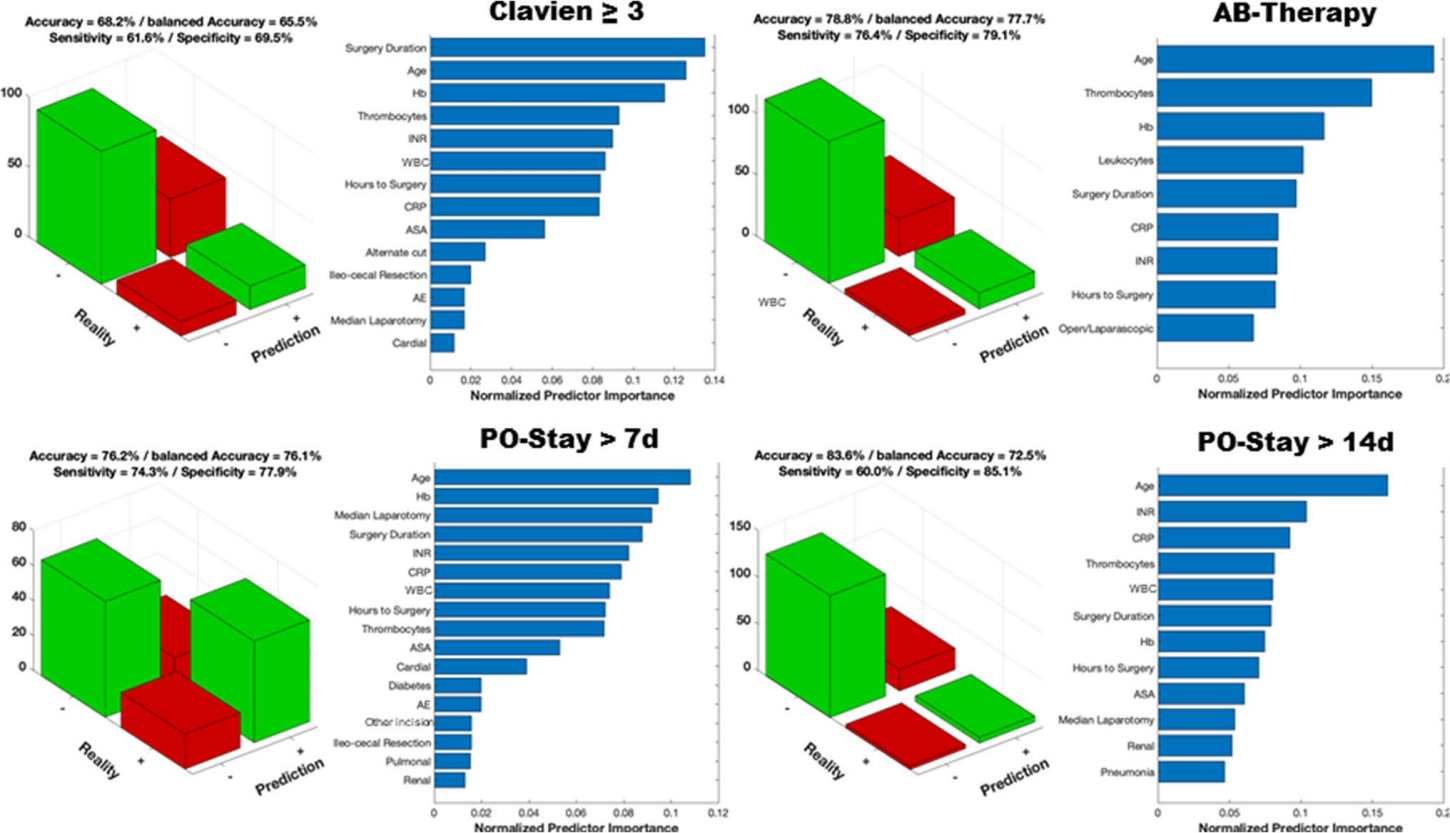
Fourfold table outcome parameter (Clavien, AB-Therapy, Hospital Stay)

The need for intensive care could be predicted with an accuracy of 77% (sensitivity: 78%, specificity: 77%). The most important predictor variables were age, ASA, cardiac disease, and CRP value. A longer stay on ICU (> 24 h) could be predicted with an accuracy of 88% (sensitivity: 88%, specificity: 88%). The important predictor variables were cardiac disease and ASA. Complications measured by Clavien-Dindo > 3 were predictable in new cases with an accuracy of 68% (sensitivity: 62%, specificity: 70%) and the most important predictor variables were age, duration of the operation, and pre-OP hemoglobin. Re-operation after initial appendectomy could be predicted with an accuracy of 74% (sensitivity: 48%, specificity: 77%), based on age and duration of the operation as the most important predictors. The occurrence of surgical site infection was predictable with an accuracy of 66% (sensitivity: 66%, specificity: 66%; important predictor variables: “age” and “duration of the operation”) and the need of oral antibiotic therapy after discharge was predictable with an accuracy of 79% (sensitivity: 76%, specificity: 79%; important predictor variables: “age” and “pre-OP thrombocytes”). The duration of hospitalization was predictable within the model for more than 7 days of hospital stay (accuracy of 76%; sensitivity: 74%, specificity: 78%; important predictor variables: “age” and pre-OP “hemoglobin”) and more than 15 days of hospital stay (accuracy of 84%; sensitivity: 60%, specificity: 85%; important predictor variables: “age” and pre-OP “INR”). All predictor variables and accuracy plots are illustrated in detail in Figures 1 and 2.

Rare complications that occurred in <2% in the examined population (mortality, fascial dehiscence, insufficiency of the stump, need for open abdomen) were not further evaluated due to the lack of adequate training samples.

## Discussion

In this study, we analyzed 163 patients with perforated appendicitis in order to develop and establish a machine learning prediction model for postoperative outcome based on a random forest classifier with stratified subsampling. Appendectomy due to any form of appendicitis is one of the most common emergency procedures worldwide and it is performed in all levels of hospital care, from rural to university hospitals [7, 8]. Postoperative complications of non-complicated (non-perforated) appendicitis are rare [7], which is why we focused on perforated cases in this model. A clinical implication for the prediction of postoperative complications can be an early transfer of patients to higher levels of care. This is especially important in case of need for intensive care treatment. Our future goal is to enable prediction of outcome of individual cases based only on preoperative data. To reach this goal, we are looking forward to a multicenter approach to train the model with more cases. With a preoperative prediction of individual cases and evaluation of a risk score, decisions of inter-hospital transfer may be made prior to surgery depending on the patients’ condition. For implementation in clinical practice, a web-based, anonymous input mask with a 3-level risk score system (low, moderate, high risk) would be desirable. In “new” preoperative cases, the prediction model will deal with “right-sided peritonism with suspicion of complicated appendicitis”. Our step-up approach is first to use further perioperative data prospectively and in a second step to fill in only the preoperative date with a feedback evaluation. If the evaluation has been successfully completed, a third step can be achieved for the first clinical application in a clinical trial.

With respect to the aim of the study, we could for the first time establish a reliable prediction model of outcome parameters for new, individual cases with perforated appendicitis. One of the most important outcome parameters in the short-term postoperative course not only for the patient but also for the planning capability of intensive care beds is certainly the necessity and duration of an intensive care stay. In our cohort, the overall rate of intensive care treatment was 26% and need for an ICU treatment was predictable in new patients with an accuracy of 77%. Of course, this rate depends on the age structure of the patient population and also the associated pre-existing conditions. Tiwari et al. describe an ICU treatment rate from 3 up to 50%, depending on the severity of illness of the patients [9]. This goes in line with our predictor variables for ICU treatment: age, ASA, and cardiac disease.

A conceivable application of the prediction model in this context would be the consideration of an early transfer to hospitals of a higher care level in the case of an expected (longer) intensive care stay, especially for basic care hospitals.

Postoperative complication as a general marker of outcome measured in the Clavien-Dindo classification (> 3) was 15% in our population and could be predicted with an accuracy of 68% in our study. In a large cohort study with data from the American College of Surgeons National Surgical database by Sood et al., the cumulated grades III-V complication rate was between 2.5 and 5% [10]. The age structure with “age” as the most important predictor of morbidity in our model was comparable in both our study and the comparison cohort of Sood et al., at 37 and 38 years respectively. However, in our cohort, 7% of patients underwent a re-operation due to initial pronounced peritonitis (i.e., a so-called planned lavage). We counted these planned lavages as Clavien-Dindo 3b complications and therefore this might explain the higher rate of severe complications in our cohort.

The length of hospital stay reflects the severity of current and pre-existing medical conditions and is often prolonged by perioperative complications. Second, hospitalization duration is not only an important parameter for estimated length of convalescence but also meaningful in terms of costs to the health care system, especially costs due to absences from work [9]. In studies of So et al. and Katsuno et al., the length of hospital stay in patients with perforated appendicitis was between 5 ± 2 and 15 ± 8 days [11, 12]. This data goes in line with 9 ± 6 days of postoperative stay in our cohort. Particularly for the reasons mentioned above, longer stays are to be given special focus (>15 days). These could be predicted with an accuracy of 84% and the main predictor variable “age” reflects the influence of pre-existing medical conditions very well.

In several studies, the time interval from hospital admission to surgery is mentioned as a risk factor for postoperative complications [13–15]. However, in a large cohort study of Almström et al. in children, in-hospital preoperative delay was no independent risk factor for postoperative complications [8]. Alore et al. also conclude on basis of their data of 112,122 patients that all appendectomies within 48 h after hospital admission are performable without increasing complications [16]. In line with these studies, the predictor “hours to surgery” was not a main predictor regarding the outcome in our study.

As a major limitation of our study, we want to highlight the monocentric and retrospective approach of our cohort study. Notwithstanding this limitation and the small case number, we could establish a robust prediction model for postoperative outcome after perforated appendicitis. We are looking forward to enlarge this model to other emergency procedures e.g., cholecystectomies.

## Conclusion

Here we demonstrate for the first time a robust machine learning-based prediction model of postoperative outcomes of patients with perforated appendicitis on the basis of a retrospective cohort study. This model is able to predict health care system-related parameters like intensive care treatment. It is therefore a conceivable application tool with important clinical implications for the consideration of an early transfer from basic care hospitals to hospitals of a higher care level in the case of an expected longer intensive care stay.

## Supplementary Information

Below is the link to the electronic supplementary material.

Supplementary file1 (PDF 339 KB)

## Data Availability

The datasets analyzed during the current study are available from the corresponding author on reasonable request. The study was approved by the local ethics committee (EK 127/16). All procedures were in accordance with the ethical standards of the institutional research committee and with the 1964 Helsinki declaration and its later amendments.
